# Inactivated *Klebsiella pneumoniae* Induces Metabolic and Hematopoietic Reprogramming to Promote Trained Immunity and Heterologous Antibacterial Protection

**DOI:** 10.3390/vaccines14040300

**Published:** 2026-03-27

**Authors:** Xiang Cheng, Shaoqiong Huang, Zhidong Hu, Xiaoyong Fan

**Affiliations:** 1 Shanghai Institute of Infectious Disease and Biosecurity, Fudan University, Shanghai 200032, China; 23211020007@m.fudan.edu.cn; 2 Shanghai Public Health Clinical Center, Fudan University, Shanghai 201508, China; 24211300002@m.fudan.edu.cn (S.H.); huzhidong@fudan.edu.cn (Z.H.)

**Keywords:** trained immunity, *Klebsiella pneumoniae*, metabolic reprogramming, hematopoietic stem and progenitor cells, heterologous antibacterial protection

## Abstract

Background: Infections caused by multidrug-resistant bacteria and inadequate vaccine coverage against opportunistic pathogens highlight the need for interventions that broadly and durably enhance host defense beyond antigen-specific adaptive immunity. Trained immunity, driven by metabolic and epigenetic reprogramming of innate immune cells, has been predominantly characterized using Bacille Calmette–Guérin and β-glucan, whereas its induction by Gram-negative bacteria remains poorly defined. To address this gap, we aimed to determine whether heat-killed *Klebsiella pneumoniae* (HK Kp) induces trained immunity through metabolic and hematopoietic reprogramming to confer heterologous antibacterial protection. Methods: HK Kp-trained murine bone marrow-derived macrophages and HK Kp-immunized C57BL/6 mice were employed to interrogate functional, metabolic, and transcriptomic reprogramming in vitro, hematopoietic progenitor remodeling in vivo, and protective efficacy against systemic *Salmonella* Typhimurium and *Staphylococcus aureus* infection. Results: HK Kp-trained macrophages showed markedly enhanced IL-1β secretion across all restimulation conditions, stimulus-dependent amplification of TNF-α responses, increased phagocytosis, and improved intracellular control of *S. typhimurium*, together with sustained upregulation of the glycolytic enzymes-encoding genes *Hk2* and *Pfkfb3*. Transcriptomic profiling revealed extensive reprogramming enriched in glycolysis/gluconeogenesis and hematopoietic cell lineage pathways. In vivo, HK Kp immunization shifted bone marrow stem/progenitor compartments toward a myeloid-biased state. HK Kp-trained mice challenged with lethal *S. typhimurium* or *S. aureus* exhibited less weight loss, improved survival rates, and reduced bacterial burdens. Conclusions: Inactivated *K. pneumoniae* orchestrates metabolic and hematopoietic reprogramming to establish enhanced innate immune responsiveness and confer heterologous protection in murine *S. typhimurium* and *S. aureus* sepsis models, supporting its potential as a potent inducer of trained immunity. These findings establish HK Kp-based trained immunity as a promising strategy for combating multidrug-resistant and vaccine-evading pathogens.

## 1. Introduction

Bacterial infections remain a major cause of morbidity and mortality worldwide despite substantial advances in antibiotics and vaccination [1]. The rise of multidrug-resistant organisms [2], together with the limited efficacy of current vaccines against many opportunistic pathogens [3,4], underscores the need for alternative strategies capable of enhancing host resistance in a broad-spectrum and durable manner. In this context, the concept of trained immunity has emerged as a paradigm shift in our understanding of host defense [5,6], challenging the traditional view that immunological memory is an exclusive property of the adaptive immune system. Trained immunity refers to the long-term functional reprogramming of innate immune cells and their progenitors following a primary stimulus, resulting in heightened or qualitatively altered responses upon secondary, often heterologous, challenges [7,8,9]. This phenomenon is driven by persistent epigenetic and metabolic rewiring [10,11] and underlies the nonspecific protective effects observed with certain vaccines and microbial products [12,13].

Most mechanistic insights into trained immunity have been derived from studies employing prototypical stimuli such as Bacille Calmette–Guérin (BCG) or β-glucan in models focusing on monocytes and macrophages [14,15,16]. These studies have established a central role for metabolic remodeling [5,17], particularly the shift toward increased glycolysis, in supporting enhanced cytokine production and antimicrobial effector functions [18,19]. More recently, compelling evidence has emerged demonstrating that trained immunity can be established at the level of bone marrow hematopoietic stem and progenitor cells (HSPCs) [8,20,21,22], leading to sustained alterations in myelopoiesis and the generation of innately primed myeloid cells [23]. However, it remains incompletely understood to what extent clinically relevant bacterial species, especially Gram-negative opportunistic pathogens, can act as potent inducers of trained immunity, and how their signals are integrated across cellular metabolism and hematopoietic lineage programs to confer heterologous protection against subsequent infections.

*Klebsiella pneumoniae* is a Gram-negative encapsulated bacterium that has become a leading cause of hospital- and community-acquired infections, including pneumonia, sepsis, and liver abscess [24,25], and is notorious for its propensity to acquire multidrug resistance, with carbapenem-resistant strains designated a critical-priority pathogen by the WHO carrying crude mortality rates of 40–58% in bloodstream and ICU-acquired infections [26]. While live-attenuated or inactivated *K. pneumoniae* preparations have been considered as vaccine candidates [27,28,29], their capacity to act as systemic “trainers” of innate immunity has not been explored. Mechanistically, heat-inactivation preserves the structural integrity of *K. pneumoniae*, thereby maintaining a composite repertoire of PAMPs comprising lipopolysaccharide (LPS), lipoproteins, peptidoglycan, and capsular polysaccharides. These PAMPs are capable of concurrently engaging multiple pattern recognition receptors (PRRs), including TLR4, TLR2, and NOD-like receptors [30,31]. The simultaneous activation of this broad PRR network may, in turn, orchestrate qualitatively distinct programs of intracellular metabolic reprogramming and hematopoietic remodeling.

We hypothesized that inactivated *K. pneumoniae* can imprint a trained immunity phenotype in macrophages, reprogram bone marrow HSPCs in vivo, and thereby endow the host with broad antibacterial protection against phylogenetically distinct pathogens. To test this hypothesis, we used heat-killed *Klebsiella pneumoniae* (HK Kp) as a model stimulus to investigate how a clinically relevant Gram-negative bacterium induces trained immunity. We established an in vitro training system in murine bone marrow-derived macrophages (BMDMs) demonstrating that HK Kp induces a canonical trained immunity phenotype, which subsequently confirmed this response in vivo through analysis of hematopoietic stem and progenitor cell composition and evaluated the protective effects in systemic infection models. Our findings reveal that HK Kp-immunized mice display enhanced resistance to both Gram-negative *Salmonella* typhimurium (*S. typhimurium*) and Gram-positive *Staphylococcus aureus* (*S. aureus*) infections, with improved survival and reduced bacterial burdens. Taken together, these results identify inactivated *K. pneumoniae* as a potent inducer of trained immunity and highlight the potential of leveraging defined inactivated bacterial stimuli to develop broadly protective, trained immunity-based interventions against diverse bacterial pathogens.

## 2. Materials and Methods

### 2.1. Preparation and Sterility Verification of Heat-Killed K. pneumoniae

HK Kp (ATCC 13883) was prepared by incubating bacterial suspensions at 65 °C for 1 h. Complete heat inactivation was verified by sterility testing: aliquots of HK Kp were plated on LB agar plates and incubated at 37 °C for 48 h; preparations were used only when no colony growth was detected. Sterility was additionally confirmed by inoculating aliquots into LB broth and monitoring for turbidity over 48 h at 37 °C.

### 2.2. Generation of Bone Marrow-Derived Macrophages and In Vitro Training Protocol

Following the protocol for analyzing trained immunity in murine BMDMs as previously described, bone marrow cells were isolated from the femurs and tibias of 5–6-week-old female C57BL/6 mice under sterile conditions [32]. Flushed bone marrow cells were passed through a 70 μm cell strainer and cultured for 5 days in complete culture medium, defined as high-glucose DMEM (Gibco, Waltham, MA, USA) supplemented with 10% (*v*/*v*) heat-inactivated fetal bovine serum (FBS; Gibco), 20 μg/mL gentamicin, and 50 ng/mL recombinant murine M-CSF (PeproTech, Cranbury, NJ, USA) at 37 °C in a humidified 5% CO_2_ incubator, with half of the culture medium replaced on day 3. On day 5, adherent cells were harvested and seeded into 48-well plates at 1 × 10^5^ cells per well, followed by stimulation with HK Kp or an equal volume of PBS for 24 h. After training, cells were washed with prewarmed PBS and cultured for an additional 5 days in fresh high-glucose DMEM containing 10% FBS and 25 ng/mL M-CSF, with a half-medium change on day 3. BMDMs were then restimulated with LPS (50 ng/mL), Pam3Cys (10 μg/mL), or heat-killed *S. typhimurium* (HK Sal, MOI = 1) for 24 h, following established protocols for trained immunity induction [33]. Culture supernatants were collected for cytokine quantification by ELISA, and cells were lysed in TRIzol reagent (TaKaRa, Kusatsu, Shiga, Japan) for subsequent RNA extraction and quantitative real-time PCR (qRT-PCR) analysis.

### 2.3. Evaluation of Cell Viability

The direct cytotoxic potential of HK Kp on macrophages was assessed through CCK-8 colorimetric analysis (Yeasen, Shanghai, China). BMDMs were plated in 96-well culture vessels and directly stimulated with HK Kp at varying multiplicities of infection (MOI: 0, 0.01, 0.1, 1, 5, and 10) for 24 h in complete culture medium. After 24 h of incubation, cells were exposed to CCK-8 substrate for an additional 4 h period. The resulting color intensity was quantified spectrophotometrically at an optical density of 450 nm. Cell viability was calculated as the percentage of viable cells in HK Kp-stimulated groups relative to untreated controls.

### 2.4. Enzyme-Linked Immunosorbent Assay (ELISA)

Concentrations of TNF-α, IL-1β, and IL-6 in culture supernatants were quantified using uncoated ELISA kits (Thermo Fisher Scientific, Waltham, MA, USA) according to the manufacturer’s instructions. Briefly, 96-well high-binding plates were coated overnight at 4 °C with the respective capture antibodies diluted in coating buffer. After three washes with PBST (PBS containing 0.05% Tween-20), plates were blocked with 1× blocking buffer for 1 h at room temperature. Following another wash, cell culture supernatants or cytokine standards (100 µL per well) were added and incubated for 2 h at room temperature. Plates were then washed five times with PBST and incubated for 1 h with the corresponding detection antibodies, followed by a 30 min incubation with HRP-conjugated secondary antibodies. After a final wash, TMB substrate was added for color development, and the reaction was stopped with 2 M H_2_SO_4_. Absorbance was measured at 450 nm using a microplate reader, and cytokine concentrations were calculated from standard curves.

### 2.5. Quantitative Real-Time PCR

Total RNA was extracted from BMDMs using TRIzol reagent in accordance with the manufacturer’s protocol. One microgram of total RNA was reverse-transcribed into cDNA using a commercial reverse transcription kit (Vazyme Biotech, Nanjing, China). Quantitative real-time PCR was performed on a 7500 Real-Time PCR System (Applied Biosystems, Foster City, CA, USA) using SYBR Green qPCR reagents (Invitrogen, Carlsbad, CA, USA). Technical replicate wells were set up for each sample, and all experiments included at least six independent biological replicates. Relative mRNA expression levels were calculated using the 2^−ΔΔCt^ method with *Hprt1* as the housekeeping reference gene and are presented as log2 fold change relative to untrained control cells. Primer sequences used for qRT-PCR are listed in Table S1.

### 2.6. Phagocytosis and Intracellular Killing Assay

BMDMs were seeded in 48-well plates at 1 × 10^5^ cells per well and infected with *S. typhimurium* (prepared at a concentration of 1.67 × 10^6^ CFU/mL in complete culture medium and added at 300 µL/well) at an MOI of 5 for 1 h at 37 °C in complete medium. After infection, extracellular bacteria were removed by washing the cells three times with pre-warmed PBS. To assess phagocytic uptake (0 h time point), cells were immediately lysed in PBS containing 0.067% SDS for 5 min at room temperature. Cell lysates were serially diluted in PBS and plated on LB agar, and colony-forming units (CFUs) were enumerated after approximately 24 h of incubation at 37 °C. To evaluate intracellular killing (4 h time point), infected cells were cultured for an additional 4 h in complete medium after removal of extracellular bacteria and then lysed, diluted, and plated as described above. Phagocytic capacity was expressed as intracellular CFU at 0 h, and the bacterial inhibition rate was calculated as [1 − (CFU4 h/CFU0 h)] × 100%.

### 2.7. Animals and Immunization

Female specific pathogen-free C57BL/6 mice (4–6 weeks old) were purchased from Huachuang Xinnuo Medical Technology Co., Ltd. (Shanghai, China). All animal experiments were approved by the Institutional Animal Care and Use Committee and conducted in accordance with the regulations of the Laboratory Animal Ethics Committee regulations at Shanghai Public Health Clinical Center. Mice were immunized by intraperitoneal injection of 200 µL PBS containing HK Kp at a dose equivalent to 1 × 10^7^ CFU of live bacteria; control mice received 200 µL PBS. A booster immunization with the same preparation was administered 1 week after the first injection, followed by a 2-week resting period before subsequent infection and analysis.

### 2.8. RNA-Seq Sequencing and Analysis

BMDMs subjected to in vitro training were divided into control and HK Kp-trained groups, with three independent biological replicates per group. Briefly, BMDMs were stimulated with HK Kp or an equal volume of PBS for 24 h, then washed and cultured for an additional 5 days in fresh high-glucose DMEM containing 10% FBS and 25 ng/mL M-CSF to allow development of the trained phenotype. Following this 5-day resting period, cells were washed twice with pre-chilled PBS and lysed in TRIzol reagent; lysates were stored at −80 °C until total RNA extraction. Total RNA was extracted, purified, and quality-controlled by OE Biotech (Shanghai, China) according to standard procedures, and only samples with an RNA integrity number (RIN) ≥ 7.0 were used for library preparation and sequencing. RNA-seq libraries were constructed using commercial kits and sequenced on an Illumina high-throughput platform with paired-end reads. After quality control, clean reads were aligned to the mouse reference genome (10 mm) to obtain gene-level read counts, and differential expression analysis was performed using DESeq2 (R package, version 1.40.1). Differentially expressed genes were defined as those with an adjusted *p* value (Padj) < 0.05 and an absolute log2 fold change > 1. Differentially expressed genes were further subjected to Kyoto Encyclopedia of Genes and Genomes (KEGG) pathway enrichment analysis and gene set enrichment analysis (GSEA). All bioinformatic analyses were carried out using standard enrichment settings by OE Biotech (Shanghai, China).

### 2.9. Flow Cytometry Analysis

Single-cell suspensions of bone marrow were prepared from the femurs and tibias of HK Kp-immunized or control mice. A total of 5 × 10^6^ cells per well were dispensed into U-bottom 96-well plates. After washing with PBS, cells were stained at room temperature with BV510 live/dead viability dye (BD Pharmingen, San Diego, CA, USA) for 15 min. Cells were then washed with staining buffer (PBS containing 2% FBS) and incubated with Fc receptor-blocking reagent (anti-CD16/32; BD Pharmingen) for 20 min at 4 °C in the dark. Subsequently, 50 µL of a premixed surface antibody cocktail was added, and cells were stained for 20 min at 4 °C in the dark, followed by a final wash and filtration through a 70 µm cell strainer. Data were acquired on an LSRFortessa (BD Biosciences, San Jose, CA, USA) or CytoFLEX S (Beckman Coulter, Brea, CA, USA) flow cytometer and analyzed using FlowJo v10 software (FlowJo LLC, version 10.8.1).

### 2.10. Antibodies

The following fluorochrome-conjugated anti-mouse monoclonal antibodies were used: anti-CD3ε-FITC (clone 145-2C11; BD Pharmingen, San Diego, CA, USA), anti-CD45R-FITC (clone RA3-6B2; BD Pharmingen, San Diego, CA, USA), anti-Ly6G/Ly6C-FITC (clone RB6-8C5; BD Pharmingen, San Diego, CA, USA), anti-CD11b-FITC (clone M1/70; BD Pharmingen, San Diego, CA, USA), anti-Ter119-FITC (clone Ter119; BD Pharmingen, San Diego, CA, USA), anti-IgM-FITC (clone RMM-1; BioLegend, San Diego, CA, USA), anti-CD34-APC (clone RAM34; BD Pharmingen, San Diego, CA, USA), anti-c-Kit-APC-Cy7 (clone 2B8; BioLegend, San Diego, CA, USA), anti-Sca-1-PE-Cy7 (clone D7; BD Pharmingen, San Diego, CA, USA), anti-CD150-BV421 (clone Q38-480; BD Pharmingen, San Diego, CA, USA), anti-Flt3-PE (clone A2F10.1; BD Pharmingen, San Diego, CA, USA), and anti-CD48-PerCP-Cy5.5 (clone HM48-1; BD Pharmingen, San Diego, CA, USA).

### 2.11. Mouse Infection and Bacterial Burden Quantification

Mice were intraperitoneally injected at week −3 and week −2 with HK Kp or an equal volume of control buffer. At week 0, a subset of mice was challenged intraperitoneally with 200 CFU *S. Typhimurium* to establish a systemic *Salmonella* infection model, while another group was challenged intravenously with 5 × 10^7^ CFU *S. aureus* ATCC 25923. At predetermined time points after infection, mice were euthanized and, under sterile conditions, livers and spleens were aseptically collected from both models and homogenized in a defined volume of sterile PBS. Tissue homogenates were subjected to 10-fold serial dilutions, and appropriate dilutions were plated on the corresponding selective or non-selective agar plates. After overnight incubation at 37 °C, visible colonies were counted, and bacterial burdens were calculated and expressed as log10 CFU per organ.

### 2.12. Statistical Analysis

All data are presented as mean ± SEM unless otherwise indicated. Sample sizes, defined as independent biological replicates, are specified in the figure legends. Comparisons between two groups were performed using two-tailed unpaired Student’s *t* tests. For experiments involving more than two groups or multiple factors, one-way or two-way ANOVA followed by appropriate post hoc tests (Tukey’s or Sidak’s multiple comparisons test) was applied, as indicated in the figure legends. Survival curves were analyzed using the Kaplan–Meier method and compared using the log-rank (Mantel–Cox) test. Statistical analyses were conducted using GraphPad Prism (version 9.0; GraphPad Software). *p* values < 0.05 were considered statistically significant.

## 3. Results

### 3.1. Heat-Killed Klebsiella pneumoniae Induces a Trained Immunity Phenotype in BMDMs In Vitro

**Figure 1:**
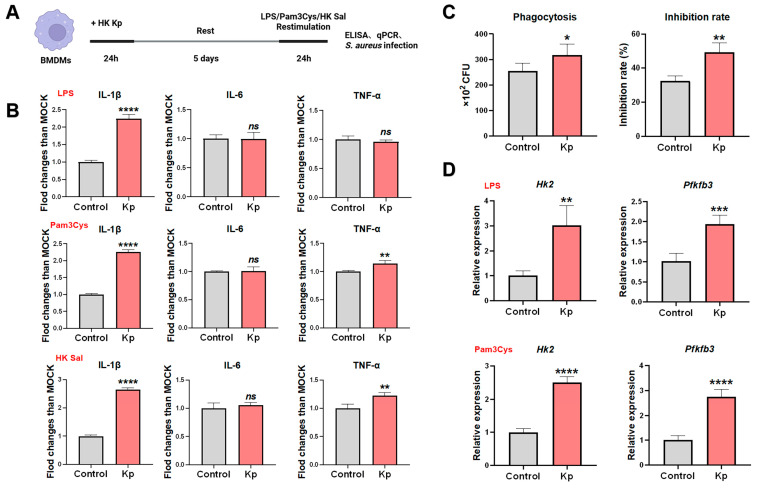
**HK Kp induces a trained immunity phenotype in BMDMs.** (**A**) In vitro training protocol. BMDMs were incubated for 24 h with medium alone (control) or HK Kp (MOI = 5), washed and rested for 5 d, and then restimulated with LPS (50 ng/mL), Pam3Cys (10 μg/mL), or HK Sal (MOI = 1) for 24 h. (**B**) After the training and resting period, BMDMs were restimulated with LPS, Pam3Cys, or HK Sal as indicated, and IL-1β, IL-6, and TNF-α concentrations in culture supernatants were quantified by ELISA and expressed as fold change relative to mock-trained controls. (**C**) Following the training and resting period, BMDMs were infected with *Salmonella*, and intracellular CFU were determined at 0 h and 4 h to evaluate phagocytic capacity and bacterial inhibition rate. (**D**) BMDMs were trained with medium or HK Kp, rested, and restimulated with LPS or Pam3Cys for 12 h; mRNA expression of the glycolytic enzymes *Hk2* and *Pfkfb3* was measured by qRT-PCR and normalized to control cells. Data are presented as mean ± SEM (*n* = 5 technical replicates per group); * *p* < 0.05, ** *p* < 0.01, *** *p* < 0.001, **** *p* < 0.0001; ns, not significant.

To determine whether HK Kp can induce a trained innate immune phenotype in vitro, we established a macrophage training system using murine BMDMs. BMDMs were stimulated with culture medium alone or HK Kp for 24 h, washed, and rested for 5 d, and they were subsequently restimulated for 24 h with LPS, Pam3Cys, or HK Sal Culture supernatants were collected for cytokine quantification and phagocytic/bactericidal activity, and glycolysis-related gene expression were assessed in parallel (Figure 1A). Importantly, HK Kp training did not compromise BMDM viability, as confirmed by CCK-8 assay showing comparable cell survival between trained and mock-treated controls (Figure S1A).

HK Kp-primed BMDMs displayed a marked increase in IL-1β secretion across all three restimulation conditions, whereas IL-6 production remained largely unchanged. By contrast, TNF-α responses were stimulus-dependent, being significantly augmented upon Pam3Cys and HK Sal restimulation but comparable to those of control cells following LPS challenge (Figure 1B). Dose-dependent experiments employing varying multiplicities of infection (MOI) of HK Sal further corroborated these findings: IL-1β demonstrated substantial fold-increases relative to mock-trained controls in a MOI-dependent manner (Figure S1B), while IL-6 and TNF-α showed more variable MOI-dependent responses (Figure S1C,D). This cytokine profile indicates that HK Kp training does not simply amplify global inflammatory output but instead establishes a trained immunity state characterized by reinforced IL-1β production with stimulus-restricted enhancement of TNF-α.

Functionally, HK Kp-trained BMDMs exhibited improved control of *Salmonella* infection: compared with control macrophages, trained cells contained more intracellular CFU at the onset of infection (0 h), consistent with increased phagocytic uptake, yet showed a more pronounced decline in CFU after 4 h, indicative of superior intracellular killing and/or bacterial growth inhibition (Figure 1C). At the metabolic level, HK Kp-trained and rested BMDMs restimulated with LPS or Pam3Cys for 12 h displayed significantly elevated mRNA expression of the glycolytic enzyme-encoding genes *Hk2* and *Pfkfb3* (Figure 1D), suggesting that acquisition of the trained immunity phenotype is accompanied by sustained activation of glycolytic pathways that support the enhanced effector functions.

### 3.2. Transcriptomic Analysis Reveals Metabolic and Hematopoietic Reprogramming in HK Kp-Trained Macrophages

**Figure 2:**
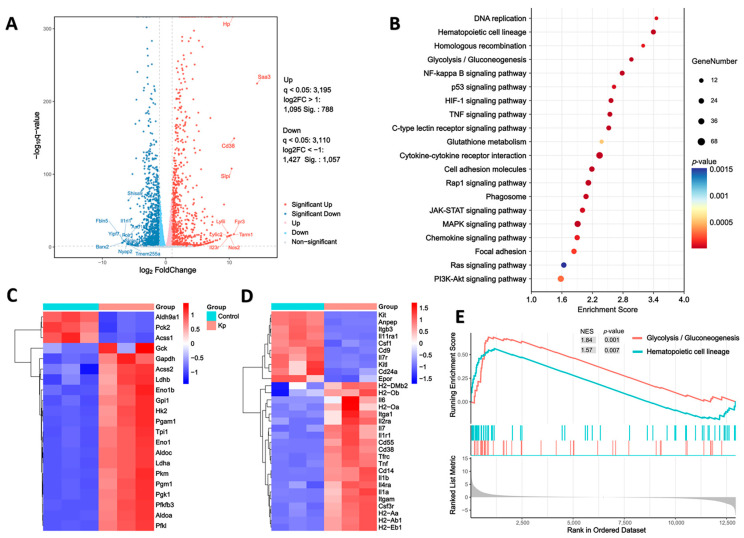
**Transcriptomic profiling of BMDMs trained with HK Kp.** (**A**) Volcano plot depicting differentially expressed genes between HK Kp-trained and control BMDMs. Significantly upregulated or downregulated genes were identified using DESeq2 with an adjusted *p* value (Padj) < 0.05 and |log2 fold change| > 1. (**B**) KEGG pathway enrichment analysis of differentially expressed genes, shown as a dot plot indicating enrichment score, gene number, and *p* value for each pathway. (**C**) Heatmap of core enrichment genes within the glycolysis/gluconeogenesis pathway. (**D**) Heatmap of core enrichment genes within the hematopoietic cell lineage pathway. (**E**) GSEA plots for glycolysis/gluconeogenesis and hematopoietic cell lineage pathways based on the ranked transcriptome. RNA-seq was performed on *n* = 3 independent biological replicates per group, and KEGG and GSEA analyses were conducted using standard enrichment settings.

To dissect the molecular underpinnings of HK Kp-induced trained immunity, we performed RNA-seq on HK Kp-trained and control BMDMs. Principal component analysis (PCA) confirmed distinct clustering of HK Kp-trained and control BMDMs, indicating substantial transcriptional divergence between the two groups (Figure S2A). The resulting volcano plot revealed extensive transcriptional remodeling, with numerous genes significantly differentially expressed following HK Kp training (Figure 2A). KEGG pathway enrichment analysis showed that these differentially expressed genes were markedly enriched in several key metabolic and immune-related pathways, with glycolysis/gluconeogenesis and hematopoietic cell lineage pathways being particularly prominent (Figure 2B). Heatmaps of core enrichment genes further demonstrated sustained upregulation of multiple rate-limiting enzymes within the glycolysis/gluconeogenesis pathway in HK Kp-trained BMDMs, accompanied by a coordinated reorganization of hematopoietic cell lineage-associated gene expression (Figure 2C,D). Notably, pathway-specific heatmaps revealed coordinated reprogramming of pattern recognition receptor signaling: toll-like receptor signaling pathway genes displayed distinct expression patterns in trained macrophages (Figure S2B), while NOD-like receptor pathway genes and downstream NF-κB signaling cascade components exhibited marked reorganization (Figure S2C,D), collectively reflecting a fundamentally rewired immune sensing and signal transduction architecture. Consistently, GSEA confirmed positive enrichment of glycolysis/gluconeogenesis and hematopoietic cell lineage signatures in HK Kp-trained cells (Figure 2E). Together, these transcriptomic data indicate that HK Kp-induced trained immunity is tightly coupled to intracellular metabolic rewiring and may additionally operate through modulation of hematopoietic lineage programs, thereby preconfiguring a heightened state of innate immune responsiveness.

### 3.3. HK Kp Immunization Remodels Bone Marrow Hematopoietic Stem and Progenitor Cell Lineages In Vivo

**Figure 3:**
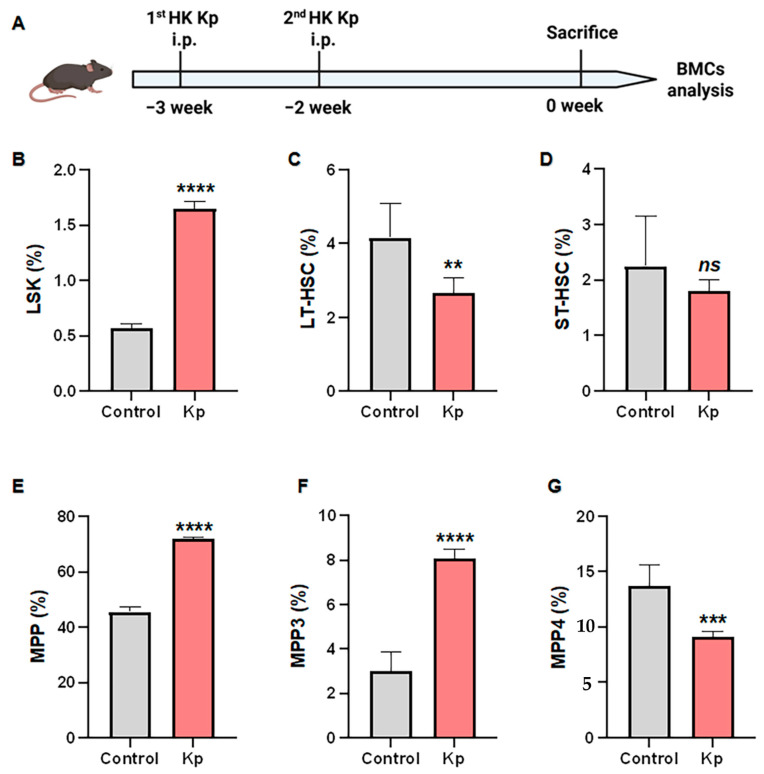
**HK Kp immunization reshapes hematopoietic stem and progenitor cell compartments in the bone marrow.** (**A**) Schematic of the in vivo immunization protocol: mice received two intraperitoneal injections of HK Kp at week −3 and week −2, followed by bone marrow cell (BMC) collection and analysis at week 0. (**B**) Frequency of LSK (Lin^−^Sca-1^+^c-Kit^+^) cells within Lin^−^ BMCs. (**C**) Proportion of LT-HSCs within the LSK compartment. (**D**) Proportion of ST-HSCs within the LSK compartment. (**E**) Frequency of MPPs within LSK cells. (**F**) Proportion of MPP3 cells within the MPP population. (**G**) Proportion of MPP4 cells within the MPP population. Data are presented as mean ± SEM (*n* = 6 mice per group); ** *p* < 0.01, *** *p* < 0.001, **** *p* < 0.0001; ns, not significant.

To investigate the hematopoietic basis of HK Kp-induced trained immunity, we established an in vivo immunization model. Mice received intraperitoneal injections of HK Kp or control buffer at week −3 and week −2, and bone marrow cells were harvested at week 0 for flow cytometric analysis (Figure 3A). The gating strategy for bone marrow hematopoietic stem and progenitor cell subsets is shown in Figure S3. Compared with controls, HK Kp-immunized mice exhibited a significantly altered frequency of LSK (Lin^−^Sca-1^+^c-Kit^+^) cells within the Lin^−^ bone marrow compartment, indicating a global remodeling of the hematopoietic stem and progenitor cell pool (Figure 3B). Within the LSK subset, the relative proportions of long-term hematopoietic stem cells (LT-HSCs) and short-term hematopoietic stem cells (ST-HSCs) were also significantly shifted, suggesting that HK Kp immunization perturbs both the composition and balance of the stem cell pool (Figure 3C,D). Furthermore, within the LSK compartment, the frequency of multipotent progenitors (MPPs) and the distribution of their functional subsets were markedly reorganized: the overall abundance of MPPs differed substantially between HK Kp-immunized and control mice, and both the myeloid-biased MPP3 and lymphoid-biased MPP4 subsets displayed a pronounced redistribution within the MPP population (Figure 3E–G). Collectively, these findings demonstrate that HK Kp immunization induces systematic reprogramming of bone marrow hematopoietic architecture, biasing the landscape toward enhanced myeloid responsiveness.

### 3.4. HK Kp-Induced Trained Immunity Enhances Host Defense Against Systemic Salmonella Typhimurium Infection

**Figure 4:**
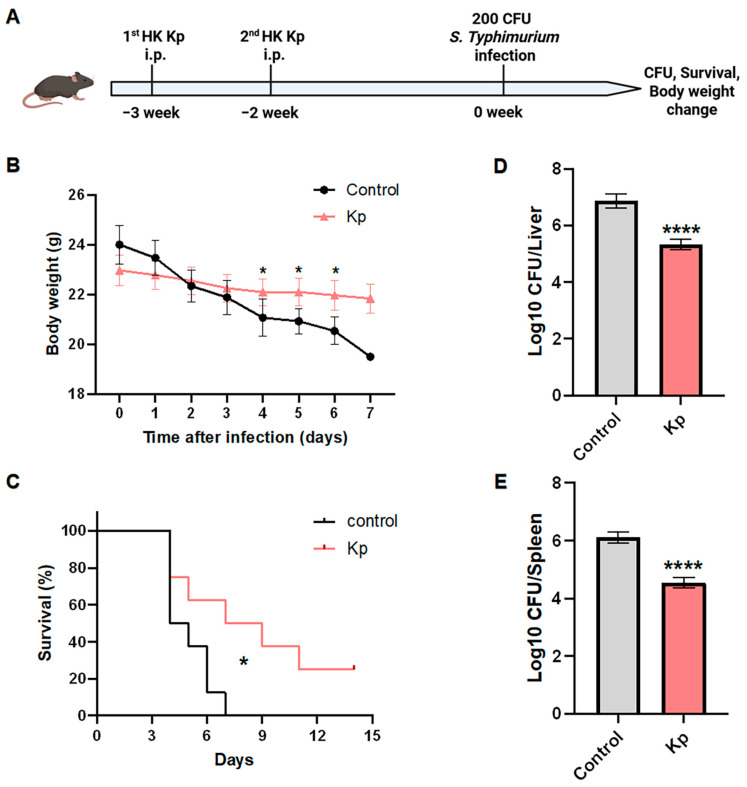
**HK Kp–induced trained immunity protects mice from systemic *Salmonella* infection.** (**A**) Schematic of the in vivo experimental design. Mice were immunized intraperitoneally with HK Kp or control buffer at week −3 and week −2, followed by infection with 200 CFU *S. typhimurium* at week 0. Body weight, survival, and bacterial burdens were subsequently assessed. (**B**) Changes in body weight over time after *Salmonella* infection in control and HK Kp-trained mice (*n* = 8). (**C**) Kaplan–Meier survival curves of control and HK Kp-trained mice following infection (*n* = 8). (**D**) Bacterial loads in the liver, expressed as log10 CFU per liver, at the indicated time point after infection (*n* = 5). (**E**) Bacterial loads in the spleen, expressed as log10 CFU per spleen, at the indicated time point after infection (*n* = 5). Data are presented as mean ± SEM; * *p* < 0.05, **** *p* < 0.0001.

To evaluate the protective effect of HK Kp-induced trained immunity against Gram-negative bacterial infection in vivo, we established a systemic *S. Typhimurium* infection model in immunized mice. Mice were immunized intraperitoneally with HK Kp or control buffer at week −3 and week −2 and subsequently challenged intraperitoneally with 200 CFU *S. Typhimurium* at week 0. Body weight, survival, and tissue bacterial burdens were then monitored longitudinally (Figure 4A). Compared with control animals, HK Kp-trained mice exhibited significantly attenuated weight loss (Figure 4B), indicative of reduced overall disease severity. Consistently, Kaplan–Meier survival analysis revealed a significantly improved survival rate in HK Kp-trained mice than in controls (Figure 4C), demonstrating that HK Kp-induced trained immunity markedly improves survival outcomes after lethal *Salmonella* infection. Bacterial burden measurements further corroborated this protective effect: at predefined time points after challenge, liver bacterial loads (log10 CFU per liver) were significantly lower in HK Kp-trained mice than in controls, and splenic bacterial burdens were likewise reduced (Figure 4D,E). Taken together, these results demonstrate that HK Kp-induced trained immunity promotes efficient clearance of systemic *S. Typhimurium* infection in vivo.

### 3.5. HK Kp-Induced Trained Immunity Confers Heterologous Protection Against Staphylococcus Aureus Infection

**Figure 5:**
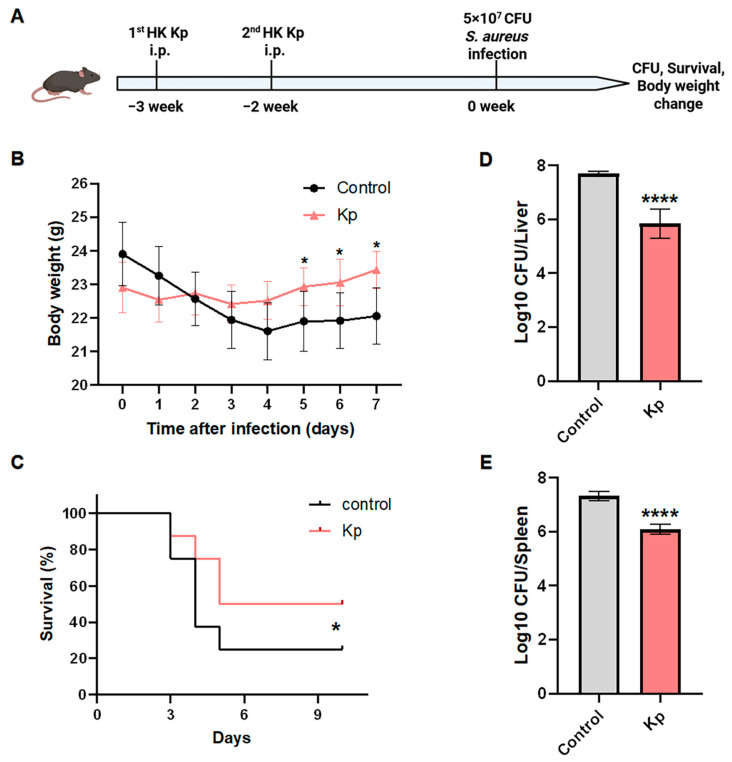
**HK Kp-induced trained immunity confers cross-protection against *S. aureus* infection.** (**A**) Schematic of the in vivo experimental design. Mice were immunized intraperitoneally with HK Kp or control buffer at week 3 and week −2, followed by intravenous infection with 5 × 10^7^ CFU *S. aureus* at week 0. Body weight, survival, and bacterial burdens were subsequently monitored. (**B**) Changes in body weight over time after *S. aureus* infection in control and HK Kp-trained mice (*n* = 8). (**C**) Kaplan–Meier survival curves of control and HK Kp-trained mice following *S. aureus* challenge (*n* = 8). (**D**) Bacterial loads in the liver, expressed as log10 CFU per liver, at the indicated time point after infection (*n* = 5). (**E**) Bacterial loads in the spleen, expressed as log10 CFU per spleen, at the indicated time point after infection (*n* = 5). Data are presented as mean ± SEM; * *p* < 0.05, **** *p* < 0.0001.

To further assess the heterologous cross-protective capacity of HK Kp-induced trained immunity, we challenged immunized mice with *S. aureus*, a phylogenetically distinct Gram-positive pathogen. Mice were immunized intraperitoneally with HK Kp or control buffer at week −3 and week −2 and subsequently challenged intravenously with 5 × 10^7^ CFU *S. aureus* at week 0. Body weight, survival, and organ bacterial burdens were then monitored longitudinally (Figure 5A). Following *S. aureus* challenge, HK Kp-trained mice exhibited significantly attenuated weight loss and more rapid weight recovery compared with control animals (Figure 5B), indicating reduced systemic inflammation and tissue damage. Consistently, survival analysis revealed that HK Kp training markedly improved survival under high-dose *S. aureus* infection (Figure 5C), supporting a cross-pathogen protective effect of HK Kp-induced trained immunity during Gram-positive bacterial challenge. In line with these outcome measures, bacterial burden analysis showed that, at predefined time points after infection, *S. aureus* loads in the liver (log10 CFU per liver) were significantly lower in HK Kp-trained mice than in controls, and splenic bacterial burdens were likewise reduced (Figure 5D,E). These findings indicate that the trained state induced by HK Kp provides heterologous cross-protection spanning diverse bacterial pathogens.

## 4. Discussion

This study demonstrates that inactivated preparations of the clinically relevant Gram-negative opportunistic pathogen *K. pneumoniae* can induce a prototypical trained immunity phenotype by jointly reprogramming cellular metabolism and hematopoiesis, thereby markedly enhancing host defense against heterologous bacterial infections in vitro and in vivo. Previous work has established that trained immunity generally relies on augmented glycolysis and remodeling of hematopoietic programs, which together provide a sustained functional “memory” basis for innate immune cells [8,23]. Within this conceptual framework, our data identify inactivated *K. pneumoniae* as a clinically relevant bacterial stimulus capable of effectively driving reprogramming along this metabolic-hematopoietic axis.

In vitro, the robust and uniform enhancement of IL-1β secretion across all restimulation conditions, together with stimulus-restricted amplification of TNF-α, improved phagocytosis, and sustained upregulation of the glycolytic genes *Hk2* and *Pfkfb3*, indicates that HK Kp establishes a qualitatively reprogrammed, rather than a globally hyperinflammatory, trained state, in line with the canonical view that trained immunity depends on glycolysis-driven metabolic rewiring [34,35,36].

Notably, genome-wide transcriptomic profiling additionally revealed significant enrichment of hematopoietic cell lineage pathways alongside glycolysis/gluconeogenesis, and GSEA confirmed positive enrichment of both gene sets in trained cells. The coordinated reorganization of hematopoietic lineage-associated genes suggests that HK Kp-induced programs act not only at the level of terminal effector cells but also reflect, or foreshadow, deeper remodeling at the hematopoietic lineage level. Together with previous reports of “central trained immunity” imprints in bone marrow HSPCs [37,38,39], the concurrent enrichment of metabolic and lineage signatures observed here offers new clues as to how trained immunity extends from intracellular metabolic restructuring to long-term memory at the level of hematopoiesis.

In vivo, HK Kp immunization globally remodeled bone marrow HSPC architecture—shifting LSK frequency, perturbing LT-HSC/ST-HSC balance, and redistributing MPP3/MPP4 subsets toward a myeloid-biased state. These findings are highly concordant with the enrichment of hematopoietic lineage pathways in the transcriptome and support the notion that HK Kp acts at the level of HSPCs to establish a central trained immunity–like state that continuously supplies pre-activated myeloid cells, thereby maintaining an elevated baseline of innate immune responsiveness at the organismal level.

Nevertheless, the precise signaling pathways by which peripheral training stimuli are translated into reprogramming of hematopoietic progenitors remain to be elucidated. Prior in vitro work has shown that the single cytokine IL-1β is sufficient to impose a stable trained immunity imprint on human hematopoietic progenitor cells [40], providing strong evidence that inflammatory mediators can directly educate HSPCs. In light of our observation that IL-1β production is robustly increased across multiple restimulation conditions in HK Kp-trained BMDMs, together with the HSPC lineage remodeling detected in vivo, the temporal and phenotypic concordance of these findings collectively suggests that IL-1β is a plausible candidate mediator linking peripheral macrophage training to bone marrow hematopoietic reprogramming. Future studies leveraging neutralizing antibodies or genetic ablation of the IL-1β/IL-1 receptor (IL-1R) axis in the HK Kp training model will be instrumental in testing whether IL-1β indeed serves as a key signaling hub that relays information from the effector periphery back to the hematopoietic “center” in clinically relevant bacteria-induced trained immunity.

At the functional level, HK Kp-induced trained immunity conferred robust heterologous protection in both *S. Typhimurium* and *S. aureus* systemic infection models, spanning evolutionarily distant Gram-negative and Gram-positive pathogens. This broad-spectrum protective profile echoes clinical and epidemiological observations that vaccines such as BCG exert non-specific anti-infective effects [41,42,43,44].

Compared with classical trained immunity inducers such as BCG and β-glucan, HK Kp shares key mechanistic features. Specifically, Quintin et al. demonstrated that β-glucan reprograms monocytes via Dectin-1-driven glycolytic rewiring and histone modifications [5], while Kaufmann et al. showed that BCG educates bone marrow HSCs through epigenetic remodeling to generate long-lived innately primed myeloid progeny [8]. Similarly, HK Kp enhances glycolysis and remodels bone marrow HSPC compartments. However, as an intact inactivated Gram-negative bacterium, HK Kp simultaneously presents LPS, lipoproteins, peptidoglycan, and capsular polysaccharides—a composite PAMP repertoire that may engage a broader PRR network involving concurrent activation of TLR4, TLR2, and NOD-like receptors [30,31]—potentially endowing the trained response with greater plasticity in cytokine profiles and functional outputs. This notion is supported by the stimulus-restricted amplification of TNF-α observed in the present study. Specifically, this “stimulus-spectrum-restricted” training pattern, whereby IL-1β is broadly enhanced but TNF-α is selectively amplified, suggests that HK Kp induces fine-tuned functional reprogramming rather than generalized hyperinflammation, which could, in principle, offer advantages in limiting immunopathology.

Several limitations should be acknowledged. First, while our transcriptomic data suggest metabolic and lineage reprogramming, direct evidence of epigenetic modifications at specific gene loci is lacking, which is essential for establishing a bona fide trained immunity mechanism. Second, the causal link between HSPCs reprogramming and the observed peripheral protection remains correlative; adoptive transfer or lineage-tracing experiments are needed to demonstrate that trained HSPCs directly give rise to functionally enhanced macrophages. Finally, the safety profile of systemic HK Kp immunization, particularly regarding LPS-mediated endotoxicity and potential inflammatory tissue damage, necessitates rigorous toxicological evaluation before clinical consideration.

Future work integrating epigenomic and multi-omics profiling with human cell-based models will be indispensable for establishing the translational relevance of HK Kp-induced trained immunity and for evaluating whether such innate immune reinforcement could ultimately be harnessed to reduce infectious complications in settings where conventional antibiotic and vaccine-based strategies remain insufficient.

## 5. Conclusions

In conclusion, our complementary in vitro and in vivo data confirm the stated hypothesis: inactivated *K. pneumoniae* functions as a potent inducer of trained immunity through coordinated metabolic and hematopoietic reprogramming, conferring significant heterologous protection against lethal *S. Typhimurium* and *S. aureus* challenge. Elucidating the trained immunity potential of inactivated *K. pneumoniae* could expand its immunological utility beyond antigen-specific adaptive responses, positioning it as a dual-modality agent capable of mobilizing both arms of host defense and informing the development of broadly protective strategies against multidrug-resistant bacterial pathogens.

## Data Availability

The original contributions presented in this study are included in the article. Further inquiries can be directed to the corresponding author.

## Supplementary Materials

The following supporting information can be downloaded at: https://www.mdpi.com/article/10.3390/vaccines14040300/s1, Figure S1. In vitro assessment of HK Kp-induced training in bone marrow-derived macrophages (BMDMs). (A) CCK-8 viability assay showing the viability of BMDMs after HK Kp training, expressed as a percentage of the mock-treated controls. (B–D) Cytokine expression following restimulation with heat-killed *Salmonella* (HK Sal) at different MOIs in HK Kp-trained BMDMs. Specifically, IL-1β expression (B), IL-6 expression (C), and TNF-α expression (D) was shown respectively, all expressed as fold changes relative to mock-trained controls. Data are presented as mean ± SEM (n = 5 technical replicates per group); ** *p* < 0.01, **** *p* < 0.0001; ns, not significant. Figure S2. Transcriptomic analysis reveals signaling pathway reprogramming in HK Kp-trained BMDMs. (A) Principal component analysis (PCA) of RNA-seq data comparing HK Kp-trained and control BMDMs. (B) Heatmap of Toll-like receptor (TLR) signaling pathway genes. (C) Heatmap of NOD-like receptor (NOD2) signaling pathway genes. (D) Heatmap of NF-κB signaling pathway genes. RNA-seq was performed on n = 3 independent biological replicates per group. Heatmaps show z-score normalized expression values; red indicates upregulation and blue indicates downregulation. Figure S3. Flow cytometry gating strategy for identification of hematopoietic stem and progenitor cell subsets in bone marrow. Sequential gating: single cells → live cells → lineage-negative (Lin-) cells → LSK (Lin^−^Sca-1^+^c-Kit^+^) cells → long-term hematopoietic stem cells (LT-HSCs)/short-term hematopoietic stem cells (ST-HSCs)/multipotent progenitors (MPPs) → myeloid-biased MPP3 and lymphoid-biased MPP4 subsets. This gating strategy was applied to bone marrow samples from HK Kp-immunized and control mice for the analyses shown in Figure 3. Table S1. Sequences of primers used for qRT-PCR analysis of mouse *Hk2*, *Pfkfb3* and *Hprt1*.
