# Supplementary material for: Inactivated Klebsiella pneumoniae Induces Metabolic and Hematopoietic Reprogramming to Promote Trained Immunity and Heterologous Antibacterial Protection
Source: Vaccines (Basel). 2026 Mar 27;14(4):300. doi: 10.3390/vaccines14040300 (PMC13120435; doi:10.3390/vaccines14040300)
Supplement: Supplementary file 1 [file vaccines-14-00300-s001.zip › Supplementary materials.pdf]

Inactivated *Klebsiella pneumoniae* Induces Metabolic and Hematopoietic Reprogramming to Promote Trained Immunity and Heterologous Antibacterial Protection

Xiang Cheng [1], Shaoqiong Huang [2], Zhidong Hu [2], Xiao-Yong Fan [1, 2, *]

[1] Shanghai Institute of Infectious Disease and Biosecurity, Fudan University, Shanghai 200032, China;
[2] Shanghai Public Health Clinical Center, Fudan University, Shanghai 201508, China;

Supplementary Figures

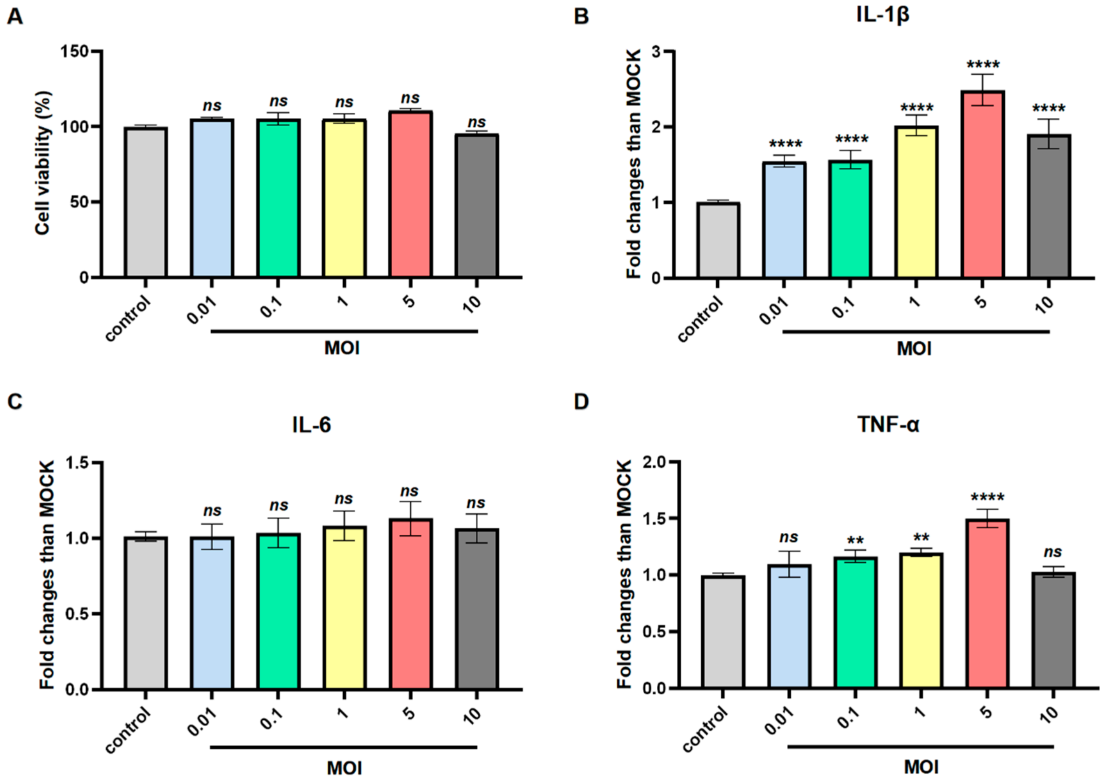

Figure S1. In vitro assessment of HK Kp-induced training in bone marrow-derived macrophages (BMDMs). (A) CCK-8 viability assay showing the viability of BMDMs after HK Kp training, expressed as a percentage of the mock-treated controls. (B–D) Cytokine expression following restimulation with heat-killed Salmonella (HK Sal) at different MOIs in HK Kp-trained BMDMs. Specifically, IL-1β expression (B), IL-6 expression (C), and TNF-α expression (D) was shown respectively, all expressed as fold changes relative to mock-trained controls. Data are presented as mean ± SEM (n = 5 technical replicates per group); **P<0.01, ****P<0.0001; *ns*, not significant.

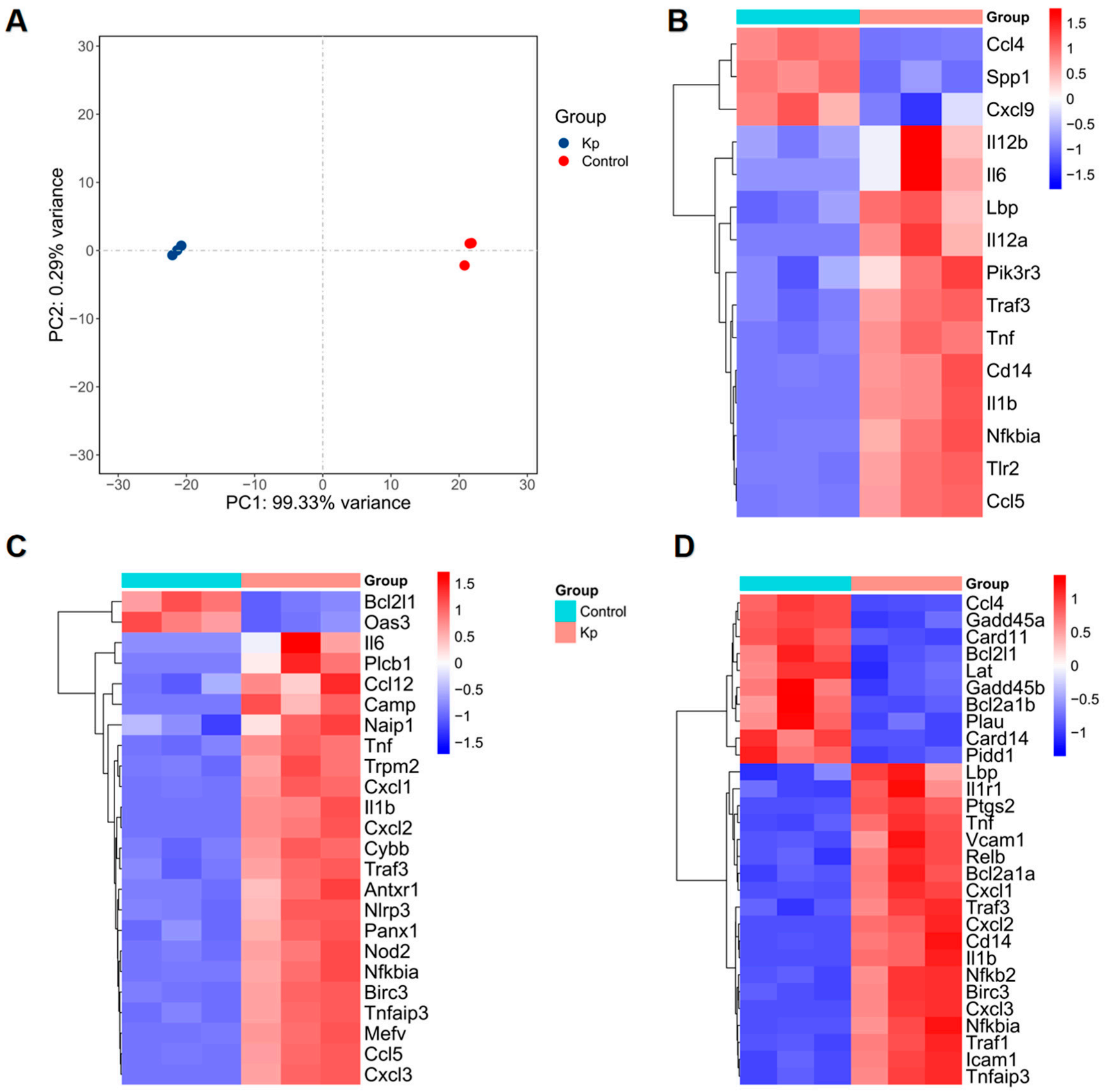

Figure S2. Transcriptomic analysis reveals signaling pathway reprogramming in HK Kp-trained BMDMs. (A) Principal component analysis (PCA) of RNA-seq data comparing HK Kp-trained and control BMDMs. (B) Heatmap of Toll-like receptor (TLR) signaling pathway genes. (C) Heatmap of NOD-like receptor (NOD2) signaling pathway genes. (D) Heatmap of NF-κB signaling pathway genes. RNA-seq was performed on n = 3 independent biological replicates per group. Heatmaps show z-score normalized expression values; red indicates upregulation and blue indicates downregulation.

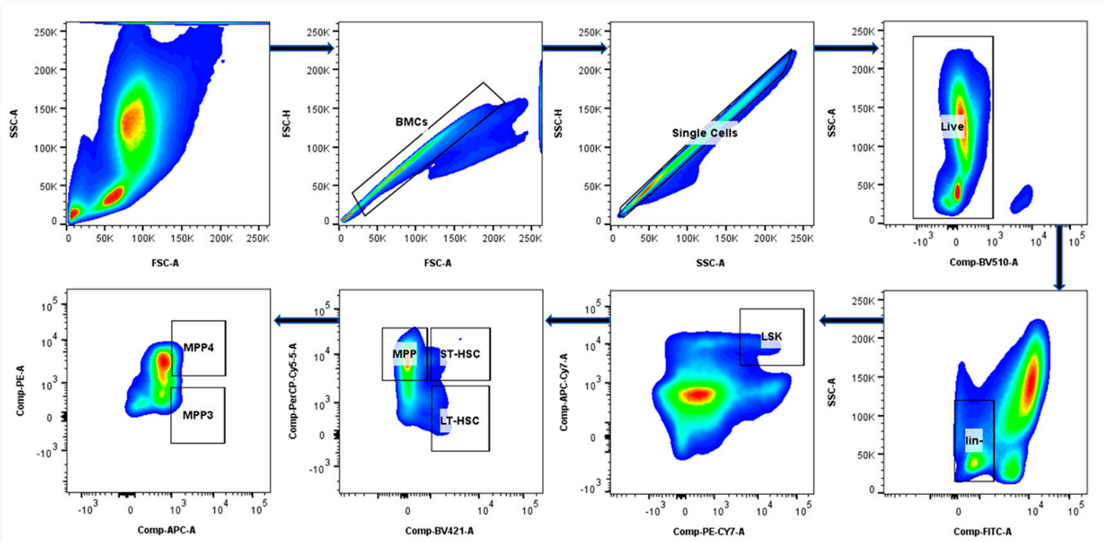

Figure S3. Flow cytometry gating strategy for identification of hematopoietic stem and progenitor cell subsets in bone marrow. Sequential gating: single cells → live cells → lineage-negative (Lin-) cells → LSK ($Lin^{-}Sca\text{-}1^{+}c\text{-}Kit^{+}$) cells → long-term hematopoietic stem cells (LT-HSCs)/short-term hematopoietic stem cells (ST-HSCs)/multipotent progenitors (MPPs) → myeloid-biased MPP3 and lymphoid-biased MPP4 subsets. This gating strategy was applied to bone marrow samples from HK Kp-immunized and control mice for the analyses shown in Figure 3.

Gene	Forward primer (5'–3')	Reverse primer (5'–3')
Hk2	TGATCGCCTGCTTATTCACGG	AACCGCCTAGAAATCTCCAGA
Pfkfb3	CGGGAGAGGTCAGAGAACATGAA	GGCCTCGAGAAGATGAGCAG
Hprt1	TCAGTCAACGGGGGACATAAA	GGGGCTGTACTGCTTAACCAG

Table S1. Sequences of primers used for qRT-PCR analysis of mouse *Hk2*, *Pfkfb3* and *Hprt1*.
